# Sexual Violence and Trauma in Childhood: A Case Report Based on Strategic Counseling

**DOI:** 10.3390/ijerph18105259

**Published:** 2021-05-14

**Authors:** Valeria Saladino, Stefano Eleuteri, Elisa Zamparelli, Monica Petrilli, Valeria Verrastro

**Affiliations:** 1 Department of Human, Social and Health Sciences, University of Cassino and Southern Lazio, 03043 Cassino, Italy; 2 Faculty of Medicine and Psychology, Sapienza University of Rome, 00178 Rome, Italy; stefano.eleuteri@gmail.com; 3 Institute for the Study of Psychotherapies, 00185 Rome, Italy; zamparelli.elisa@gmail.com; 4 Academy of Social and Legal Psychology, 00198 Rome, Italy; monicapetrilli@hotmail.it; 5 Department of Medical and Surgical Sciences, University of “Magna Graecia”, 88100 Catanzaro, Italy; valeriaverrastro@unicz.it

**Keywords:** sexual addiction, childhood sexual abuse, BDSM, distress

## Abstract

Children and adolescents are too often victims of sexual abuse and harassment. According to the World Health Organization (WHO), approximately 150 million girls and 73 million children <18 have been victims of violence and sexual exploitation during their childhood. Data show that females are more likely to be a victim of abuse and violence than males (20% vs. 5–10%). Such abuses lead to long-term psychophysical and relational consequences and victims are often afraid of asking for support from both parents and professionals. This case report shows the story of a 17-year-old adolescent, Sara, involved by her mother in a strategic counseling process, to solve BDSM-type sexual addiction (slavery and discipline, domination and submission, sadism and masochism), self-aggressive behavior, and alcohol abuse issues. The strategic counseling process is structured in 15 sessions and was based on problem-solving techniques and corrective behavioral strategies. During the sessions, it emerged that Sara had been a victim of sexual violence at the age of 6 and that she had never talked about the rape with anyone. At the age of 12, she began to experience social anxiety and shame, feelings that led her to use alcohol and seek violent sexual partners and bondage relationships. During the counseling sessions, Sara elaborated on her trauma, becoming more aware of her resources and her desires, and she learned to manage the sense of guilt and shame associated with the violence suffered, through alternative strategies. At the end of the process, Sara normalized her relationship with sex and alcohol, regaining her identity.

## 1. Introduction

### 1.1. Sexual Abuse in Childhood: Definitions, Spread, and Consequences of the Phenomenon

The World Health Organization defines “child maltreatment” as all the forms of abuse and neglect that involve children. This definition includes physical and emotional violence, sexual abuse, neglect, and exploitation. These abuses lead to damage to children’s health, impacting their development [1]. According to the fundamental rights of the European Union and the United Nations Convention on the Rights of the Child, children should be protected against all forms of violence, and adults should promote their well-being [2,3]. There are different definitions of child sexual abuse. For instance, sexual harassment can arise on a continuum of power and control, from non-contact sexual assault (such as exhibitionistic actions) to contact sexual assault (such as rape). Additionally, Internet sexual offending is included in the definitions of child sexual abuse. This category concerns the distribution, acquisition, and possession of child sexual exploitation material, child grooming, and online contact with children for gratifying sexual desire (e.g., receiving sexually explicit images or cybersex) [4]. Regarding the spread of this phenomenon, it is estimated that one billion children are a victim of some form of violence. Thus, one out of two children per year worldwide suffers from some form of violence. Furthermore, the COVID-19 pandemic has increased the risk of children being victims of violence within their families [5,6]. Indeed, social distancing and restrictions impacted the levels of stress and anxiety, reducing usual sources of support and increasing online abuse [7,8].

Our study focuses on sexual violence and sexual abuse, which means the involvement of children or teens in sexual coercion or sexual harassment. These experiences may not involve explicit violence or injury and could occur without physical contact or be experienced as observers. Sexual abuse can be divided into different categories depending on the relationship between the child and the perpetrator. Intra-familial abuse is implemented by family members, peri-familial abuse is implemented by people external to the family but who take care of the child; and extra-familial abuse involves perpetrators who are not part of the family environment [9].

Child sexual abuse is connected to several unpleasant consequences. Victims may develop mental health problems, such as affective disorders, suicidal ideas, drug or alcohol addiction, social anxiety, conduct disorder, borderline personality disorder, post-traumatic stress disorder, and eating disorders, in particular bulimia nervosa [4]. Furthermore, child sexual abuse harms the physical health of children, leading to urogenital complaints (e.g., genital pain, dysuria, genital bleeding, and incontinence problems) [10]. According to Adams et al. [11], the severity, duration, and onset of sexual abuse influence the level of depressive, anxiety, and post-traumatic stress disorder (PTSD) symptoms. Regarding gender differences, the authors found that sexual abuse produces the worst effects in females. Indeed, the early onset of sexual abuse may cause anxiety symptoms in females but not among males. In the same line, it seems that sexual abuse may determine PTSD mostly in females but not in male adolescents.

The stage of development in which children suffered from abuse (early childhood, childhood, adolescence) can influence the severity of the consequences for health. Traumatic experiences, such as violence and abuse, lived during the first few years of life have a stronger impact on the development than those experienced in another period [12]. Van Duin et al. [13] examined the impact of extra-familial sexual abuse among children under four years old and the consequences for their parents. The results show that 3% of children developed a PTSD diagnosis, 30% of them exhibited clinically significant sexual behavioral problems, while 24% of them showed internalizing problems, 27% attachment insecurity and 18% received a psychiatric disorder diagnosis. Regarding parents of children who suffered from abuse, 20% reported high levels of PTSD symptoms, with mothers reporting PTSD symptoms more often than fathers. They also suffered from feelings of guilt, shame, and anger. The authors hypothesized that the psychological treatment provided to 25% of the victims and 45% of parents mitigated the negative consequences.

Additionally, suffering from extreme abuse for a long period, having a close relationship with the perpetrator [14], and living in dysfunctional families are risk factors associated with the development of severe psychological symptoms [15]. Moreover, the risk of re-victimization is higher among children who suffered from sexual abuse compared to others. The disclosure of the trauma is hard because of feelings of shame, guilt, and intimidation by the perpetrators and the wish to not burden the family. The stigmatizing response by the social environment influences the development of shame and guilt linked to sexual victimization. This is also connected to the feeling of being blamed or judged.

These data underline the importance of educating society in understanding the consequences of sexual victimization and in supporting prompt reporting. These results might be useful in promoting therapeutic interventions to support victims and to decrease the dysfunctional cognitions of sex offenders [16].

### 1.2. Sexual Addiction and BDSM among Survivors of Childhood Sexual Abuse

Several authors have reported that 80% of people [17] who experienced child sexual abuse (CSA) developed compulsive sexual behavior and sexual addiction in adulthood. Pereira et al. [18] confirmed the relationship between childhood sexual abuse and a later disposition toward compulsive sexual behaviors. They found that sexual abuse experiences and poor family relationships during childhood enhance vulnerability to initiating and maintaining out-of-control sexual behaviors. They confirmed this association, with a prevalence in the male population that seems to be more susceptible to the development of sexual addiction and compulsion. Thus, this behavior is a transversal phenomenon that vulnerable people can use to manage intense and negative emotions related to the distress of abuse [19].

The experience of women with sexual compulsivity is intensely shame-based and difficult to deal with. The family preconditioning of abandonment in childhood emerges through inadequate care, experiences of abuse, abandonment, and the presence of other addictions, as shown by case studies analysis [20,21]. As children, these women were looking for something to ease their distress when they could not rely on their caregivers. Mostly, they use maladaptive coping mechanisms, such as compulsive masturbation, binge eating, and violent fantasies, to maintain their sanity in childhood.

According to Freud’s theory, at the basis of this behavior might be a trauma suffered by the children caused by the experience of impotence and the contact with a threatening adult [22]. This experience triggers strong anguish in the face of which the child activates a series of defenses to protect themselves, including the conversion of the trauma and identification with the aggressor. These modalities convert, to quote Stoller, “the infantile trauma into an adult triumph” [23].

Sexual atypia and paraphilias lead to reliving the traumatic experience while preserving the illusion of control and sexual gratification, which provides individuals with a false sense of power that preserves their integrity. The strong aggression and anguish distort the vision of the other, who becomes a dehumanized object. This is the mode implemented to cope with strong emotions derived from traumatic experiences [24].

Indeed, at the origin of masochism, there could be an infantile experience of passivity and annulment. In this case, the mechanism of reversal of the experience undergone is structured as a masochistic defense. In the adult re-perpetration of the trauma staged in the perversion, the person is no longer the passive victim of an executioner but the holder of control. It is the subject who asks the executioner to suffer and to be objectified. This perception gives masochistic pleasure to the person. From this point of view, the masochist’s pain is a defense against the greater and deeper pain of rejection [22].

In this way, sadomasochistic sexual practices could assume a key role in sexual trauma processing. BDSM (slavery and discipline, domination and submission, sadism and masochism) is receiving increasing attention from the scientific community. The term BDSM identifies a wide range of erotic practices between two or more consenting partners who share sexuality based on games of power, dominance, and submission from which they derive satisfaction and pleasure. Today, the BDSM phenomenon is viewed from a biopsychosocial perspective [25].

Studies show a positive correlation between BDSM interests and personality traits, adverse childhood experiences, education levels, sexual orientation, and biological indicators. The limitations of the research lie in the fact that most studies so far are only descriptive [26,27]. Some researchers have focused on better understanding the aspect of pain within a BDSM interaction [28,29], as experiencing afflicting or receiving pain is a relevant part of BDSM interaction. The result is that BDSM practitioners seem to have a higher pain threshold overall and, specifically, submissive BSDM interaction results in a constant increase in pain thresholds [28].

Further research focused on the rewarding biological mechanism associated with BDSM interaction. They found that submissive practitioners showed increased cortisol and endocannabinoid level due to the BDSM interaction, while dominant practitioners only showed increased endocannabinoid levels when the BDSM interaction was associated with power-plays [30].

### 1.3. Strategic Counseling

Strategic counseling is an intervention that refers to the theory of strategic psychotherapy and aims to reach a specific goal through techniques based on communication. Strategic counseling is efficient in modifying patients’ points of view and in promoting the solution of their issues [31,32].

Strategic counseling is effective in managing personal, relational, and working problems. One of the most important characteristics of strategic counseling is the focus on the function and the dynamic of the issue (“how my problem works”), instead of on the causes (“why I have a problem”). The focus is on the present and the future and not on the past, which represents a starting point to assess patients’ cognitions [33]. According to the process of strategic counseling, there are solutions as well as problems and these solutions are strongly related to the characteristics of the issue, akin to a dress tailored to the patient. Strategic counseling is a flexible type of counseling—it adapts to the specific problem until it leads the person to perceive the problem differently and therefore to change their behavior. The most used element of strategic counseling is communication, the so-called “strategic dialog” [34,35].

Strategic communication is characterized by a series of techniques that lead people to discover new ways of perceiving and managing problematic situations. Therefore, strategic dialogue leads to an experience of changing one’s own feelings and perceptions, modifying one’s perspective. Strategical problem solving is one of the most common techniques, which we could define as the “technology” to find solutions because of its effectiveness in finding alternatives [36,37,38].

Finding alternative solutions to a problem is not easy and leads to implementing the usual solutions, the so-called “attempted solutions”, but which turn out to be unsuccessful, only increasing the sense of inadequacy and dissatisfaction [39]. The attempted solutions have the function to maintain the problem and to create a vicious circle in which the person is psychologically trapped.

Strategic problem solving modifies the dynamics of rational linear thinking to find the solution, through stratagems of non-ordinary logic. This allows finding a solution in the present rather than an explanation in the past [38].

Therefore, strategic counseling is characterized by its flexibility and adaptability to the problem presented, since it makes use of strategies and techniques conceived and adaptable to the established purpose. Indeed, as the counseling intervention proceeds, it can be reoriented based on the observed effects. This intervention method guides clients to change their behavior, their feelings about the problem, and the perception of events, changing their perspective of observation and feelings connected to the problem. Clients experience new perceptions and discover different ways to manage and overcome difficulties. These sensations and perceptions become actions and behaviors that lead to higher individual awareness. According to this perspective, behavior change derives from a modification of perceptions that simultaneously generate a different way of conceiving and relating to reality or “to change to know” [35].

### 1.4. Strategic Behavioral Prescriptions

Prescriptions are tasks and indications that the therapist provides during the sessions. The patient must perform these tasks between sessions or during the session itself. In strategic counseling, behavioral prescriptions represent an important function, since to bring about a change one must go through concrete actions, acting on the problem even in the absence of the therapist [40]. This absence allows patients to demonstrate that they can change their situation from a concrete experience. Prescriptions can be direct, indirect, and paradoxical [41].

In the first case, these are clear indications about the actions that the patient should perform. These aim at achieving a specific and shared goal in the session. Collaborative patients with low resistance benefit from this type of prescription.

Indirect prescriptions are behavioral injunctions that hide their true goal and circumvent the individual’s resistance. These prescriptions are best suited to those who resist changing. They act persuasively through linguistic and hypnotic suggestions. The therapist shifts the patient’s attention from the problem to other elements that reduce the tension linked to the discomfort, allowing the individual to neutralize the problem.

The paradoxical prescriptions, on the other hand, provide for the use of the symptom of resistance to therapy, as actions to be voluntarily implemented or exasperated to increase the level of control perceived by the patient about a previously spontaneous situation [41].

The therapist reinforces the results obtained by the patients, redefining the situation and gratifying them [42]. Prescriptions play a key role in strategic counseling and are part of the change process as they create a bridge between the patient’s reality and the therapeutic setting.

## 2. Materials and Methods

### 2.1. Procedure

This case report illustrates the story of Sara, an Italian girl of 17 years of age. Sara suffers from BDSM-type sexual addiction, self-aggressive behavior, and alcohol abuse because she was a victim of sexual harassment when she was a child. Sara was involved by her mother in a strategic counseling program. The strategic counselor (SC) was a young woman, and the therapeutic process was divided into 15 weekly sessions which were 60 min long, as described in Table 1:

### 2.2. Ethical Statement

Sara and her mother were informed by the strategic counselor that the therapy will be part of a scientific publication. The aims, the methods, and the procedure were explained to the minor and her mother in verbal and written forms. The SC obtained the informed consent of the participants to publish the therapy in online and paper journals. The participants were aware that their sensitive data (names, places, etc.) would be subject to change to protect privacy.

The informed consent was redacted according to the Italian Deontological Code of Psychologists of the National Council of the Order of Psychologists 2020 (www.psy.it) (accessed on 10 May 2021) and was based on the following Italian legislative references: Law 633/1941 Article 96 (Protection of copyright and other rights related); Civil Code Article 10 (Abuse of the image of others); Civil Code Article 23 (Consent for personal data processing); Legislative Decree n. 196/03 Article 13 and EU Regulation 2016/679 (GDPR) Article 13 (Information on the processing of personal data).

The collected materials are kept confidential under the responsibility of the SC.

## 3. Case report: Sara’s Story

### 3.1. Assessment and Therapeutic Alliance

Sara goes to art school, she is an only child and has lived alone with her mother since her father left home when she was 10. Sara was involved in the strategic counseling process by her mother, worried about her daughter’s high-risk behaviors, such as engaging in sex with several occasional partners and binge drinking.

Although it is not her direct request, Sara immediately demonstrates a good motivation to undertake the therapeutic path. Moreover, despite her young age, Sara shows good awareness of her psychological, physical, and cognitive state, expressing her feelings and thoughts.

During the first session, the strategic counselor invites Sara’s mother to attend. However, Sara shows considerable difficulty in talking about herself in the presence of her mother, so the SC decides to be alone with Sara. In the absence of her mother, Sara discloses with the SC, although with slight difficulty, describing her problem in relations with other, especially males, and telling the SC about the trauma she experienced when she was six, namely a sexual assault by a man of 20. Sara expresses embarrassment and shame in telling of her traumatic experience, holding her head, avoiding the therapist’s gaze, and never using the word “rape”. Sara also affirms that since she was 12, she has had violent sexual intercourse and bondage relationships with peers. Furthermore, when she decides to have sex with someone, she also binge drinks alcohol. Sara’s mother is unaware of her daughter’s trauma and believes that her daughter’s destructive attitudes are part of her adolescence. Sara describes her mother as a “normal, slightly apprehensive housewife”. She considers her childhood quite happy. However, when she was very young, her mom went through a particularly intense moment of emotional distress due to numerous conflicts with her father, which is why she often had to care for her ailing mom. For this reason, Sara decided to avoid telling her mother about the sexual violence.

The SC uses the techniques of active listening and lets all the elements emerge that the girl is ready to share. At the end of the session, the SC thanks Sara for the trust and courage shown and gives the girl a task, called “my objectives”. According to the prescription, she is required to describe the personal goals which she wants to obtain by means of the therapy.

During the second session, Sara comes alone. She sits and starts to read the task without any encouragement from the SC. Sara identifies her sex life as a source of discomfort. Her sexuality is characterized by strong and uncontrollable impulses, which lead her to seek out sexual acts and physical violence. When she has these impulses, she often contacts some friends who practice BDSM, namely bondage (not professional), and she asks them to have violent sexual intercourse, after binge drinking. Her mother discovered her activity when Sara came home one night with marks and bruises all over her body. On that occasion, Sara admitted to her mother that she has a problem managing her sexuality and that she explicitly asks her partners to practice bondage and to inflict upon her asphyxiation and physical violence. The girl explains that this kind of suffering gives her the feeling of control, as she is the one who decides to feel pain and the level to which she does so. An aspect that Sara realizes in completing the prescription is that when she had homosexual intercourse, she never felt the need to suffer violence. With boys, however, she sought violence during penetration and, in particular in practicing bondage. Sara realizes that her sexual impulses never allowed her to have a relationship and she describes sex as a punishment that she uses because she thinks she does not deserve to be loved.

The SC and the patient identify the main objectives of the therapeutic process as elaborate on the trauma deriving from sexual abuse suffered in childhood, exploring sexuality without the use of alcohol or violence, and structuring a new self-image considering her desires and resources.

During the third session, the SC describes to the patient the process of trauma processing, explaining the role of repressed emotions and memories. Despite the fear of reliving her trauma, Sara shows a strong motivation to continue the process, saying that she stopped her sexual impulses after her mother discovered them. Additionally, Sara admits that during those nights in which she practiced bondage, she felt that she could have suffocated. That event impacted the perception of Sara towards her sexuality, leading her to relive the same fear she felt during the abuse she suffered. Sara is not ready to tell her mother the truth. However, she does not exclude the possibility of doing this in the future.

It appears immediately functional for Sara to have a young female figure as a therapist to project and analyze some dynamics of her behavior without fear of being judged and to gradually acquire confidence.

### 3.2. Sexual Violence-Trauma Processing and Positive New Identity Structure

The second part of the therapeutic process was focused on trauma-processing and on the possible evaluation of the dysfunctional coping strategies used by Sara to manage her negative feelings connected to the traumatic experience. First, Sara was instructed to explore the dynamics of her trauma, learning how to change the memories linked to the experience. Sara explored her body’s reaction during her processing of trauma, and she also understood what happens in talking about her sexual violence. She analyzed her physical and emotional reactions, living and exploring the situation in the therapeutic setting. The second step of the trauma-processing was to lead Sara to tell her traumatic story from multiple points of view, exploring it as if she could relive it but from the outside, as an observer. The goal was to teach Sara, progressively, how to understand the emotions connected to the trauma and be able to face them, and then live the experience as a story that is part of her life but that can no longer hurt her because it belongs to the past. To reach this goal, the SC gave to Sara a specific prescription: “imagine that you can do something to make your current condition worse, imagine the worst fantasy on your traumatic experience”. This prescription leads the patient to realize that she has control over the decision-making process about her malaise and to analyze all the attempted solutions she has implemented and continues to implement even though they are dysfunctional, to solve her problem.

At first, Sara expressed difficulty in imagining how her traumatic experience could have been worse; however, later she created alternative scenarios that saw her capable of worsening her fantasy about the past and about the present. Sara gained more control over her choices and realized that self-harm related to extreme sexuality and alcohol abuse stems from this idea of not having control over her body, as someone else has it instead. When she carries out these harmful behaviors, she allows another person to harm her, and this removes her responsibility and reduces her sense of guilt for not having protected herself during the aggression suffered as a child.

Sara analyzed her relationship with alcohol and stated that what she appreciates most is the sense of relaxation and the absence of tension. However, once this effect is over, Sara suffers a psycho-physical breakdown, sadness, and a sense of emptiness. The substance, therefore, mitigates the anxiety of the girl, who slowly undertook to reduce its use to monitor the effect that these changes in behavior have on well-being. Additionally, Sara practices bondage during sexual intercourse, and she feels very protected and accepted and can share an aspect of herself that is more complex to externalize. Sara does not share this aspect of her life with her mother and feels it is not understood. After she started the therapeutic path, however, Sara became more confident in her relationship with her body, asking her mother for advice and support.

The SC explained to the patient that she should not aim to reach a socially shared normal range, but the goal is to understand its functioning and progressively reduce everything that causes her discomfort. Bondage-related BDSM experiences are denial and defense mechanisms that Sara uses, along with alcohol, to avoid dwelling on her suffering. The two thematic areas, bondage and alcoholic binging, are intertwined during the sessions. These draw a parallel between the desire, the effects, and consequences of alcohol and the violent sexual experiences Sara sought. These two conditions have in common the associated emotions and the subsequent intense sense of emptiness and sadness. Sara now hypothesizes that she can do without the search for risky or self-harming situations and for the first time she reflects on the meaning that these actions have for her, as she said: “I thought I was in control by drinking and doing violence to me, but it was my addiction to violent sex and alcohol that had control over me”.

The SC explained to the patient the theory of the “self-fulfilling prophecy”, asking her to reflect on how she could change this prophecy. The last four sessions before the therapeutic restitution and closing phase were focused on the building of a new positive identity structure. Sara was required to learn and apply strategies to (a) monitor her psycho-physical sensations related to self-harming behaviors, alcoholic binges, and bondage, understanding the relationship with her emotions; (b) learn to respect her body more and to assume control of her decisions, passing from a passive to an active role; and (c) find alternatives to violent sexual behaviors.

To achieve these aims, the SC gave to Sara the following prescriptions:

(a) A diary of emotions, structured to analyze the type of emotion, the antecedent, thoughts, actions, strategies, and consequences and to become more curious and attentive toward her feelings and reactions.
(b) The use diaphragmatic breathing once per day, to get in touch with her body, focusing on physical needs.
(c) Dedicating some time to her sexual pleasure, identifying new activities and modalities as alternatives to violent bondage, associated with alcohol abuse.

Regarding the first prescription, during the sessions, Sara reported having monitored her emotions related to negative feelings and the need to self-harm through sex and alcohol as her coping strategies. She described some events in which she felt alone and misunderstood by her mother or friends. However, analyzing the situations, she found inconsistencies between her interpretation and the reality, understanding how often she tends to blame others for her malaise. Doing this exercise every day and illustrating to the SC all the reported events and emotions, Sara progressively recognized that she has mental patterns that lead her to perceive others as threats and to feel misunderstood and alone. Her strategy is always to make others abuse her to confirm her attribution of guilt.

Sara is now more aware of the way she uses violent sex and alcohol and the psychophysical consequences that come with it. This careful analysis of her behavior, together with the other two prescriptions, allowed the girl to get in touch with the needs of her body, to focus on physical and sexual well-being, without feeling negative emotions and guilt or punishing herself sexually with bondage. In fact, during the weeks between sessions, Sara learned to dedicate herself to the well-being of her body and explored other activities related to sexual pleasure that did not involve the use of violence, such as masturbation and petting, only with people of who she trusts. Additionally, Sara progressively talked with her mother, telling her about her progress.

The emotions during the task related to the exploration of sexuality were positive, as Sara was able to give herself something satisfying. A very interesting and adaptive sensation for the patient’s functioning is that she felt pampered and protected by herself during masturbation and by her sexual partner during petting. Sara also decided to no longer engage in sexual activities that result from negative emotions. Bondage for her will always be an aspect of her sexuality but she wants to be able to decide and not let her trauma take over.

The new ways of exploring sexuality that Sara learned required considerable effort. Sara was very brave and found a personal space in which to find her own new identity. These sensations are new for her, as Sara has never explored her body or recognized her sexual needs, if not mediated by violence. The contact with the body, now experienced positively, reduces the sense of shame.

The SC positively reinforces Sara’s need to find herself, reflecting on her resources. Sara has already found her inner space, she just has to keep feeding it in order to not fall back into the old dysfunctional strategies. To do this, Sara needs to continue with the prescription for another two weeks, in which she will explore her autonomy from the SC. Sara can contact the professional if she needs to, but she does not come to the counselor’s office for two weeks. In addition, the SC gives Sara another prescription, to add to the previous ones: “What I want when I want”. According to this behavioral task, Sara will do something that she desires to do, just to experiment with the pleasure of doing this. Sara can do more than one thing, but she must do at least one per day.

### 3.3. Therapeutic Restitution and Follow-Ups

This conclusive part of the therapeutic process aims to reinforce the positive outcomes obtained by the patient and to restitute feedback on the acquired strategies for the future. To reach these goals, the SC used the suitcase metaphor, comparing the therapeutic experience to a journey during which Sara learned some coping strategies useful to manage stressful events and to better organize her new life and identity. The patient imagines having a suitcase in which she can collect and take these strategies with her, to cope with difficulties and to maintain her outcomes.

During the last session, the SC asked Sara to describe her therapeutic process, looking to herself as an observer and pointing out the main changes and results obtained. Sara was enthusiastic to describe her improvements. She reported some actions done for the “What I want when I want” prescription, such as spending time with friends, reading books, and taking long walks. Sara did not show any negative feelings, such as shame or embarrassment, in admitting her old habits and in recounting the trauma experienced in childhood. She now is more aware and less afraid of her impulsive and destructive behaviors and aims to build a “true” identity, not being influenced by the violence suffered. Sara, however, expresses her fear of falling back into the dysfunctional behaviors and harming herself again.

The SC explains therefore that the therapy foresees two follow-up sessions, one after one month and one after three months. These sessions are aimed at monitoring Sara’s progress and helping her to maintain them over time. This perspective reassures the patient, who greets the SC with affection and gratitude. The SC also explains to Sara that in the month in which they will not see each other, she will have the sole task of using the tools learned in therapy, metaphorically opening her suitcase, and pulling out the ones that best suit the situation she must deal with.

After one month, the SC meets Sara and she tells the professional about her progress. During the month, the teenager often confronted her mother, explaining her problems and asking for help when she felt aggressive impulses. The mother responded adequately to her daughter’s requests, showing herself to be present and welcoming. Sara also tells of having opened her suitcase on several occasions and having used some strategies learned in therapy. Sara decided to continue to explore her sexuality through masturbation and petting and only with people she trusts. She did not experiment with compulsions regarding sexuality or binge drinking, and she started to practice sport (running) to take care of her body. During the session, the SC reinforced the prescription of the suitcase, adding the task to identify other tools and strategies to add to it.

Three months later, the SC conducted the last follow-up with the patient. During the session, Sara was excited to tell the SC her improvements. Indeed, she was selected to participate in a competitive run, and she also had her first sexual intercourse without the use of violence or bondage. Sara felt satisfied with her progress and she also added a tool in her suitcase: get in touch with her physical sensations. Sara started to concentrate more on her body and her physical needs. The SC compliments Sara and positively strengthens her progress. The counselor asks her to continue filling her suitcase with useful strategies and tools for her future.

## 4. Discussion

According to the definition of child sexual abuse, sexual harassment can be described on a continuum of power and control from non-contact sexual assault to contact sexual assault [4].

This phenomenon is widespread among children; indeed, one out of two children per year worldwide suffers some form of violence [43].

The recent scientific literature on sexual abuse shows that the consequences of this traumatic event jeopardize both the physical and psychological health of the individual and cause lifelong distress. The gradual emergence of symptoms following exposure to traumatic events represents a conceptual challenge for psychology and psychiatry. Indeed, child sexual abuse is associated with mental health issues, drug or alcohol addiction, and post-traumatic stress disorder [4].

This work aims to explore the possible relationship between experiences of childhood abuse and the development of pathological compulsive sexual behavior, sexual addiction and BDSM conducts. These pathological sexual behaviors are characterized by inappropriate or excessive sexual acts or cognitions that lead to subjective distress or impaired functioning.

Sadomasochistic sexual practices are receiving greater attention from the scientific community than in the past. The acronym BDSM identifies a wide range of erotic practices between two or more consenting partners who share sexuality based on games of power, dominance, and submission from which they derive satisfaction and pleasure. Risk factors are thought to include family history and childhood abuse, and it seems that compulsive sex and BDSM practice represent a functional behavior to compensate for the traumatic experiences of abuse [44].

The case report illustrated herein shows an association between sexual violence and risky behavior in adolescence. Sara is a 17-year-old adolescent who suffered sexual violence when she was six and developed a sexual addiction relating to BDSM (bondage) and binge drinking. The mother of Sara, worried about her daughter, introduces Sara to the strategic counseling process. The SC sessions were divided into 15 sessions with specific goals and prescriptions. At first, Sara revealed her story, talking of the sexual abuse and of the tendency to have violent sexual intercourse, to practice BDSM, specifically bondage, and to drink alcohol before sex. The first step of strategic counseling’s process (three sessions) was to focus on the assessment and on establishing a therapeutic alliance. Specifically, the SC created a therapeutic alliance with the patient, who learned to manage negative emotions related to the abuse. Sara associated herself with the counselor, a young woman, and established a strong relationship of trust. The patient and the counselor used this positive relationship to co-build objectives for the other steps of the therapy.

The second (five sessions) and the third steps (four sessions) were focused on sexual violence-trauma processing and had the aim of elaborating the memories related to the trauma and on building a new identity. In this phase, Sara analyzed her relationship with alcohol and BDSM. She understood the role of substance abuse and violent sex in her life. Indeed, on the one hand, alcohol and sex mitigate the feeling of anxiety and she feels invincible, but on the other hand, Sara perceives a sense of shame and guilt. During this process of evaluation of her coping strategies, Sara started to communicate with her mother, asking for support. The SC guided Sara to discover more functional strategies for her well-being. The counselor explained the theory of the “self-fulfilling prophecy” and encouraged Sarah to make a positive prophecy about herself come true. At the end of this phase of counseling, Sara learned to monitor her psycho-physical sensations related to self-harming behaviors, alcoholic binges, and bondage, understanding the relationship with her emotions, and to respect and explore her body more, assuming the control of it and being active, as well as in her sexuality. These skills led Sara to find alternatives to violent sexual behaviors.

The final step (one session) was based on the therapeutic restitution and the SC positively reinforced the outcomes of the therapeutic process and restituted feedback on the acquired strategies for the future. To do so, the SC used the metaphor of the suitcase, according to which Sara can collect the strategies she learned into this suitcase and she can open it and use them anytime she feels the need.

The SC performed the first follow-up one month after the end of the therapy and a follow-up after three months. During both follow-ups, Sara demonstrated having maintained the positive outcomes of the therapy and using her suitcase to cope with difficulties. Sara practiced sport and stopped using BDSM, violent sex, and alcohol. Today, her relationship with sexuality is based on self-eroticism and petting with people she trusts.

## 5. Conclusions

The review of the literature and the case report presented highlighted the importance of exploring the possible connection between childhood sexual abuse and the development of compulsive sexual behavior and BDSM practices in adulthood. The recognition that comes from relationships with others (partner, sexual interest, work) confirms the value of our existence. Consequently, some individuals could undertake their search for contact through the forced transition from the passive to the active role, displaying risky behavior concerning fears and life experiences.

Hypersexuality and sadomasochistic practices might compensate for the missing part of the subject’s ego. The pain of self-esteem’s loss, parental affection, or childhood omnipotence is anesthetized through perverse action and fantasy. Sexual practices based on violence and coercion in some cases allow individuals to act out their fantasy of perfection. In this act, the sadist is reunited with his/her lost omnipotent self and the masochist abandons himself/herself in the other, rediscovering the fusion condition of childhood.

This division between reality and fantasy, between loss and the denial of grief, is also reflected in the thinking of these subjects as an inability to accept different views, without considering nuances or a middle ground.

## Figures and Tables

**Table 1:** Strategic counseling sessions and objectives.

Sessions	Objectives	Prescriptions
Assessment and therapeutic alliance (3 sessions)	Creating the therapeutic alliance and learning to manage negative emotions, sense of guilt and shame.	“My objective”
Sexual violence-trauma processing (5 sessions)	Processing the trauma related memories.	“Worst fantasy on my trauma”
Positive new identity structure (4 sessions)	Identifying resources, reducing self-harm conducts (violent sexuality and binge drinking), and building new identity.	- Diaphragmatic breathing - The diary of emotions - My sexuality - “What I want when I want”
Therapeutic restitution (1 sessions)	Reinforcing positive outcomes and restitute a feedback on the acquired strategies for the future.	“The suitcase”
First Follow-up (1 sessions)	Evaluate after 1 month the therapeutic outcomes.	“Add strategies to the suitcase”
Second Follow-up (1 session)	Evaluate after 3 month the therapeutic outcomes.	“Add strategies to the suitcase”
